# Characteristics of SARS-CoV-2 Infections in Israeli Children During the Circulation of Different SARS-CoV-2 Variants

**DOI:** 10.1001/jamanetworkopen.2021.24343

**Published:** 2021-09-07

**Authors:** Ido Somekh, Michal Stein, Isabella Karakis, Eric A. F. Simões, Eli Somekh

**Affiliations:** 1 Department of Pediatric Hematology Oncology, Schneider Children’s Medical Center of Israel, Petah Tikva; 2 Sackler Faculty of Medicine, Tel Aviv University, Tel Aviv, Israel; 3 Infectious Disease and Infection Control Unit, Hillel Yaffe Medical Center, Hadera, Israel; 4 Rappaport Faculty of Medicine, Technion – Israel Institute of Technology, Haifa, Israel; 5 Epidemiology Division, Environmental Epidemiology Department, Public Health Services, Ministry of Health, Jerusalem, Ben-Gurion University in the Negev, Beer-Sheba, Israel; 6 University of Colorado School of Medicine, Aurora; 7 Department of Pediatrics, Mayanei Hayeshuah Medical Center, Bnei Brak, Israel; 8 European Pediatric Association (EPA-UNEPSA), Union of National European Pediatric Societies and Associations, Berlin, Germany

## Abstract

This cohort study compares the characteristics of infections from SARS-CoV-2 variants spreading during August to October 2020 vs the variants spreading during December 2020 to February 2021 among children in Israel.

## Introduction

Since December 2020, the SARS-CoV-2 B.1.1.7 variant has been spreading in Israel, and by January or February 2021 it quickly became the predominant circulating strain, isolated in more than 80% of cases.^1^ Concomitantly, a mass COVID-19 vaccination campaign was launched in Israel.

The aim of this study was to compare the characteristics of SARS-CoV-2 spread in children, aged 0 to 9 years, in 2 periods when different SARS-CoV-2 variants were circulating: August to October 2020, when former SARS-CoV-2 variants (mostly GH and GR clades)^2^ circulated, and December 2020 to February 2021 since the introduction of the B.1.1.7 variant in Israel.

## Methods

### 

This cohort study followed the Strengthening the Reporting of Observational Studies in Epidemiology (STROBE) reporting guideline. According to the Clinical Trials in Humans regulations published by the Israeli Ministry of Health, this study was considered exempt from institutional review board approval and did not require informed consent because it used publicly available, deidentified data.

For children aged 0 to 9 years, publicly available national daily data regarding SARS-CoV-2 polymerase chain reaction tests performed, rates of positive samples, COVID-19 incidence, and number of hospitalizations were obtained from the Ministry of Health. Data were not stratified according to race and ethnicity, and nationwide data were analyzed.^1,3^ Weekly incidence rates were adjusted for the number of tests performed^4^ (eAppendix in the Supplement). SARS-CoV-2 case investigations and contact tracing were performed by the Ministry of Health (eAppendix in the Supplement). Nonpharmacologic interventions during the study periods including vaccination campaign data are also detailed in the eAppendix in the Supplement.

During the periods of August 1 to October 2, 2020, and December 3, 2020, to February 3, 2021, the following outcomes were analyzed and compared: (1) curves of adjusted incidence were plotted and their linear regression slopes were compared; in addition, Poisson regression was used to compare differences between the 2 periods; and (2) SARS-CoV-2 transmission and hospitalization rates were also examined.

Two-tailed χ^2^ and *t* tests were used for statistical analysis, and *P* < .05 was considered statistically significant. Statistical analyses were conducted using SPSS Statistics version 25.0 (IBM Corp) from March to June 2021.

## Results

Data were analyzed for 21 615 children aged 0 to 9 years (50.9% male children) who had positive SARS-CoV-2 polymerase chain reaction tests between August 1 to October 2, 2020, and for 50 811 children aged 0 to 9 years (51.5% male children) who tested positive between December 3, 2020, and February 3, 2021. The slopes of weekly adjusted incidence curves for children aged 0 to 9 years during December to February 2021 (84.4; 95% CI, 71.1- 97.7) were significantly higher than those in August to October 2020 (39.1; 95% CI, 23.9-54.3). Rate ratio of highest to lowest weekly-adjusted incidence was higher during December 2020 to February 2021 (6.75 [95% CI, 6.3-7.2]) compared with August to October 2020 (3.62 [95% CI, 3.4-3.8]) (Figure). Likewise, the difference between the 2 periods was statistically significant following Poisson regression analysis (*P* < .001).

**Figure.**
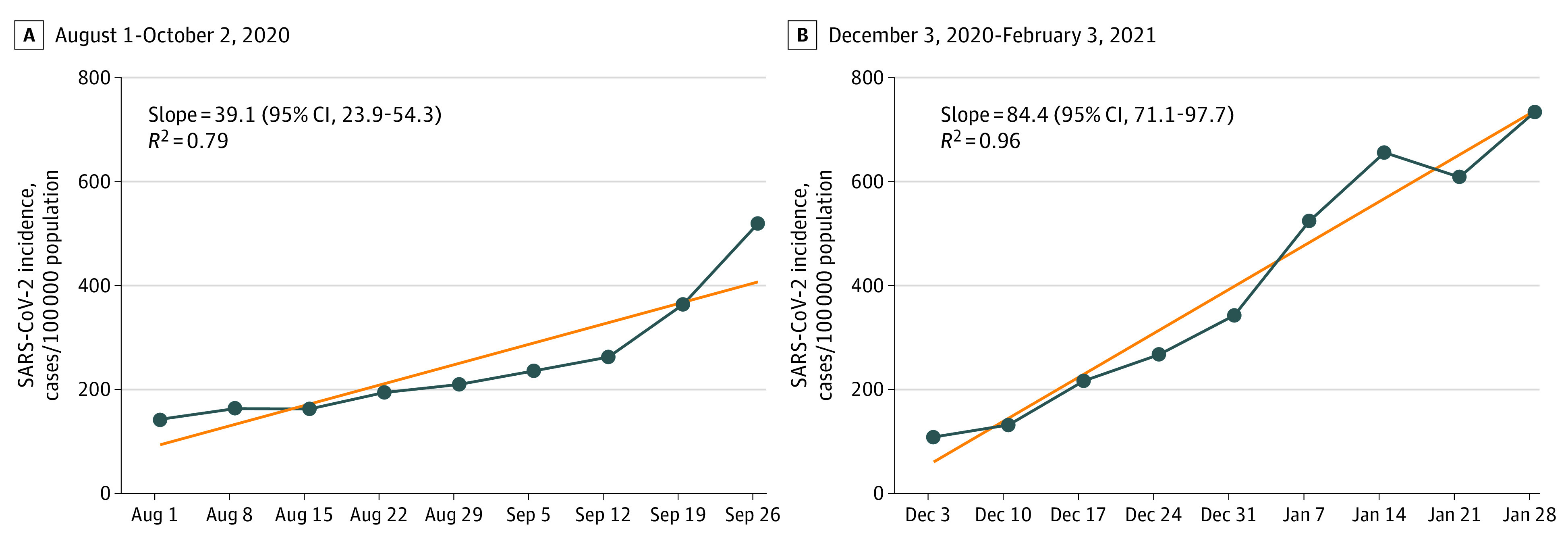
Weekly Adjusted Incidence Rate Curves in Studied Periods Weekly adjusted incidence (new SARS-CoV-2 cases/100 000 in children aged 0-9 years) are presented during the respective periods. Linear regression curves are depicted by the lines connecting the dots. Dates outlined represent day 1 of the studied week. *R*^2^ denotes the *R*^2^ value of the regression line. The slope (with 95% CI) is for the regression line. The ratio of highest to lowest weekly adjusted incidence rates was higher during December 2020 to February 2021 (6.75 [95% CI, 6.3-7.2]) compared with 3.62 (95% CI, 3.4-3.8) during August to October 2020.

Analysis of contact tracing found that during February to November 2020, 7.5% of traced secondary cases (11 770 of 156 521) were related to children aged 0 to 9 years. This rate increased significantly to 15.7% (49 257 of 313 871) during December 2020 to April 2021 (rate ratio [RR], 2.24; 95% CI, 2.20-2.29; *P* < .001).

During August to October 2020, there were 261 hospitalizations among 26 689 individuals aged 0 to 9 years diagnosed with SARS-CoV-2 (0.98%) compared with 379 hospitalizations among 72 796 new cases during December to February 2021 (0.52%). Hospitalization rates were significantly lower in the latter period (RR, 0.53; 95% CI, 0.46-0.63; *P* < .001). The percentage of hospitalized children with unfavorable outcomes (severe condition and death) out of total number of hospitalizations was not different between the 2 periods (6.9% vs 6.5% in the late and early periods respectively (early period: 6.5% [17 of 261] vs late period: 6.9% [26 of 379]; RR, 0.99; 95% CI, 0.96- 1.04).

## Discussion

These results demonstrate that SARS-CoV-2 spread more effectively and more rapidly among young children during the time of B.1.1.7 variant circulation in Israel. Transmission rates from children aged 0 to 9 years to other contacts were doubled during the time of B.1.1.7 circulation in Israel. However, hospitalization rates among children decreased. The latter finding is supported by studies in adults reporting increased contagiousness of the B.1.1.7 strain but not necessarily with increased severity.^5,6^

The B.1.1.7 variant that spread in children during December 2020 to February 2021 period remained significantly higher than during earlier spread of the GH and GR variants. Higher spread during December 2020 to February 2021 was observed despite the expected indirect mitigating effect on children of mass vaccination of adults on children. These findings illustrate the higher rates of transmission of this variant in children, highlighting the importance of making COVID-19 vaccine available for young children. Nonpharmacologic measures such as lockdown and school closure could not account for the difference in transmission since they were used during both periods. These findings suggest that health authorities in different regions should anticipate this occurrence and implement measures to reduce spread in young children both in schools and at home.

The main limitation of this study is its observational design. Because no specific sequencing was done on the samples, other factors (aside from B.1.1.7 strain) might have contributed to these findings.

## Notes

Supplement. **eAppendix.** Supplemental Methods
eReferences
