# Supplementary material for: Characteristics of SARS-CoV-2 Infections in Israeli Children During the Circulation of Different SARS-CoV-2 Variants
Source: JAMA Netw Open. 2021 Sep 7;4(9):e2124343. doi: 10.1001/jamanetworkopen.2021.24343 (PMC8424472; doi:10.1001/jamanetworkopen.2021.24343)
Supplement: Supplement. — eAppendix. Supplemental Methods eReferences [file jamanetwopen-e2124343-s001.pdf]

Supplemental Online Content

Somekh I, Stein M, Karakis I, Simões EAF, Somekh E. Characteristics of SARS-CoV-2 infections in Israeli children during the circulation of different SARS-CoV-2 variants. *JAMA Netw Open.* 2021;4(9):e2124343. doi:10.1001/jamanetworkopen.2021.24343

eAppendix. Supplemental Methods

eReferences

This supplemental material has been provided by the authors to give readers additional information about their work.

© 2021 Somekh I et al. *JAMA Network Open.*

eAppendix. Supplemental Methods:

1. **Data source**: Israel Ministry of Health has operated a Corona Surveillance Computerized Database including the results of RT-PCR tests and epidemiological investigations with mandatory reporting of laboratory confirmed SARS-CoV-2 infections and associated hospitalizations. Hospitalized cases are daily updated regarding clinical conditions and mortality. The decision whether to hospitalize children is based upon the clinical discretion of the hospitals staff.
2. **Adjustment of incidence rates**: SARS-CoV-2 age group specific weekly incidence rates were calculated and adjusted for the number of tests performed for age groups 0-9 years: incidence rate (weekly number of new cases /100,000 population of the specific age group) was multiplied by the proportion of this age group in the general population and divided by the proportion of the samples taken from individuals of this age group out of all samples obtained. [1]
3. **Epidemiological investigations**: Epidemiological investigations start when a person is identified as a COVID-19 case by a positive RT-PCR test. The collected data include age, address, gender, possible contacts, and clinical information (symptoms, chronic morbidity etc.). Public health investigators identify contacts with a positive COVID-19 case, and track them down specifically identifying temporal relationships and whether they are COVID-19 positive by an RT-PCR test. With this information it is possible to determine the estimated source of exposure and the estimated place of infection and to determine the share of

© 2021 Somekh I et al. *JAMA Network Open.*

transmission following contact with children aged 0-9 years compared to that of older cases. All epidemiological investigations are performed by officially trained and licensed investigators and all results are recorded.

4. **Non-pharmacologic measures:** Non-pharmacologic measures during the study periods include: National lockdowns (from September 18 to October 13, 2020, and from January 8 to February 7, 2021) and School closures (September 14 – November 1, 2020 and January 8 - February 15, 2021).
5. **The COVID-19 vaccination campaign:** The COVID-19 vaccination campaign in Israel was launched on December 20, 2020 and by February 3, 2021, 57% of adults were vaccinated with the first dose of BNT162b2-mRNA COVID-19 vaccine.

eReferences:

1. Somekh I, Keinan-Boker L, Shohat T, Pettoello-Mantovani M, Simões EAF, Somekh E. Comparison of COVID-19 Incidence rates before and after school reopening in Israel. JAMA Netw Open. 2021 Apr 1;4(4):e217105. doi: 10.1001/jamanetworkopen.2021.7105. PMID: 33900403.

© 2021 Somekh I et al. *JAMA Network Open.*
